# Habitat fragmentation is associated to gut microbiota diversity of an endangered primate: implications for conservation

**DOI:** 10.1038/srep14862

**Published:** 2015-10-07

**Authors:** Claudia Barelli, Davide Albanese, Claudio Donati, Massimo Pindo, Chiara Dallago, Francesco Rovero, Duccio Cavalieri, Kieran Michael Tuohy, Heidi Christine Hauffe, Carlotta De Filippo

**Affiliations:** 1 Department of Biodiversity and Molecular Ecology, Research and Innovation Centre, Fondazione Edmund Mach, Via E. Mach 1, 38010 S. Michele all’Adige, Trento, Italy; 2 Tropical Biodiversity Section, MUSE – Museo delle Scienze, Corso del Lavoro e della Scienza 3, 38123 Trento, Italy; 3 Reproductive Biology Unit, German Primate Centre, Leibniz Institute for Primate Research, Kellnerweg 4, 37077 Göttingen, Germany; 4 Department of Computational Biology, Research and Innovation Centre, Fondazione Edmund Mach, Via E. Mach 1, 38010 S. Michele all’Adige, Trento, Italy; 5 Department of Genomics and Biology of Fruit Crops, Research and Innovation Centre, Fondazione Edmund Mach, Via E. Mach 1, 38010 S. Michele all’Adige, Trento, Italy; 6 Department of Food Quality and Nutrition, Research and Innovation Centre, Fondazione Edmund Mach, Via E. Mach 1, 38010 S. Michele all’Adige, Trento, Italy; 7 IBIMET-CNR, Via Caproni 8, 50145, Firenze, Italy

## Abstract

The expansion of agriculture is shrinking pristine forest areas worldwide, jeopardizing the persistence of their wild inhabitants. The Udzungwa red colobus monkey (*Procolobus gordonorum*) is among the most threatened primate species in Africa. Primarily arboreal and highly sensitive to hunting and habitat destruction, they provide a critical model to understanding whether anthropogenic disturbance impacts gut microbiota diversity. We sampled seven social groups inhabiting two forests (disturbed vs. undisturbed) in the Udzungwa Mountains of Tanzania. While Ruminococcaceae and Lachnospiraceae dominated in all individuals, reflecting their role in extracting energy from folivorous diets, analysis of genus composition showed a marked diversification across habitats, with gut microbiota α-diversity significantly higher in the undisturbed forest. Functional analysis suggests that such variation may be associated with food plant diversity in natural versus human-modified habitats, requiring metabolic pathways to digest xenobiotics. Thus, the effects of changes in gut microbiota should not be ignored to conserve endangered populations.

Although changes in the functional diversity of the microbial community populating the gastrointestinal tract (gut microbiota) have been shown to affect development, health, fitness and evolutionary trajectory in both humans and other animals^1^,^2^,^3^,^4^,^5^,^6^,^7^,^8^,^9^,^10^, the implications of these changes for animal conservation efforts have not been addressed. A number of drivers are known to influence gut microbiota composition, including diet, physiology and evolutionary history^2^,^11^,^12^,^13^,^14^,^15^. In addition, the physical environment may also determine interspecific and interpopulation differences (e.g. as shown in fish^16^, iguanas^17^, birds^18^, bats^19^ and mice^20^).

The influence of gut microbiota homeostasis on African primate conservation is of particular interest given their threatened status. Africa’s forests are fragile environments, and changes in land use are significantly decreasing pristine forest areas, resulting in loss of unique habitats. Environmental degradation often leads to a decline in species richness of non-generalist flora and fauna, while fragmentation may also result in unviable population sizes and hence, local extinction. If habitat loss also leads to changes in gut microbiota, this could have knock-on effects on individual health, and increase the probability of such extinctions. The effect of habitat quality and degradation on gut microbiota diversity has been shown in the endangered howler monkeys by Amato *et al.*^21^, and in various primate species by McCord *et al.*^22^. However, the impact of environmental changes on metabolic capacities based on inferred gut microbiome has not been addressed elsewhere, which allow comparative analyses at individual and population levels.

Here we analyse the phylogenetic and functional diversity of the gut microbiota in a threatened endemic primate, the Udzungwa red colobus (*Procolobus gordonorum*; IUCN 2014, Version 2014.3, available at www.iucnredlist.org, accessed 5 March 2015, Supplementary Fig. S1), recognized as an important flagship species^23^. This non-human primate has populations in both large intact forest blocks and forest fragments of the Udzungwa Mountains, in the Eastern Arc Mountains of Tanzania and Kenya, a global biodiversity hotspot^24^,^25^,^26^. Thus, it provides an excellent model for investigating the effects of habitat degradation and fragmentation on the gut microbiota. Using barcoded 454 amplicon pyrosequencing of the 16S bacterial ribosomal RNA gene of faecal samples, 16 collected in Magombera (Ma) and 15 collected in Mwanihana (Mw), we hypothesized that gut microbiota composition and function would be altered in populations restricted to fragmented and degraded forests, and that these modifications would be associated with changes in the metabolic pathways at the level of the microbiome.

## Results and Discussion

**Figure 1:**
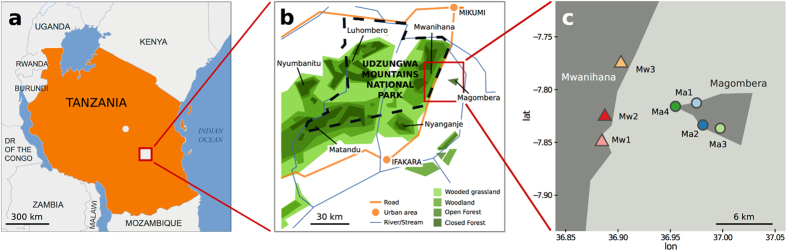
Map of the study sites in the Udzungwa Mountains. Tanzania (**a**), enlargement indicating the two selected forest blocks, Mwanihana and Magombera (**b**), sample sites for the seven groups of Udzungwa red colobus (**c**). Dashed line in (**b**) denotes the border of the Udzungwa Mountain National Park. Symbols identify forests (i.e. triangles and circles for Mwanihana and Magombera respectively), while colours identify each social group. Figure (**a**) is based on OCHA map and edited with Inkscape (www.inkscape.org); Figure (**b**) was generated with Inkscape; Figure (**c**) was generated with R (www.r-project.org) with the ggmap library and edited with Inkscape.

We described the gut microbiota of geographically isolated populations of red colobus monkey (n = 31) from a large pristine forest block and a remnant (Fig. 1; Supplementary Table S1 and Supplementary Fig. S2) in the Udzungwa Mountains of Tanzania, which differ in levels of human disturbance. The two forested areas have been separated by 10 km for at least 60 years due to human settlements and intensive agriculture. Within Ma forest, logging causes further forest loss and 40% is heavily degraded^23^,^27^. In contrast, Mw forest is within the well-protected Udzungwa Mountains National Park^28^ (Fig. 1b), established in 1992, and part of a large swathe of continuous forest with a higher plant diversity than Ma^27^,^29^,^30^.

### Gut microbiota composition of red colobus across forests

**Figure 2:**
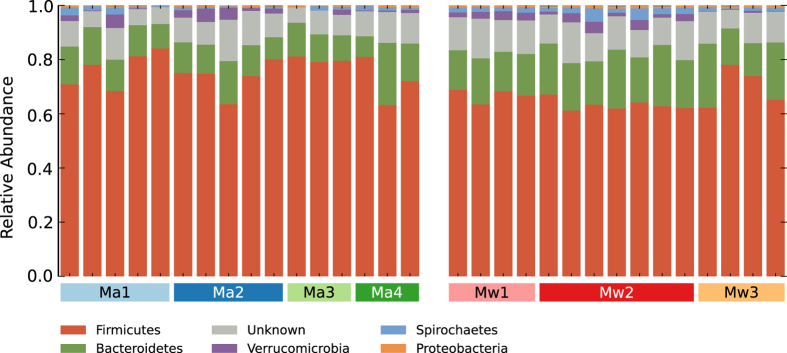
Relative abundances of the five most abundant bacterial phyla of red colobus faecal microbiota. The most abundant phyla are Firmicutes, Bacteroidetes, Verrucomicrobia, Spirochaetes and Proteobacteria of faecal microbiota in each individual among the of seven Udzungwa red colobus social groups from the Magombera (n = 16) and Mwanihana (n = 15) forests. Symbols are colour coded according to Fig. 1c.

The shared gut microbiota of red colobus living in Ma and Mw includes the most abundant Phyla, Firmicutes and Bacteroidetes, as noted in other mammals^2^ (Fig. 2, Supplementary Table S2). Interestingly, and similar to Yildirim *et al.*’s report^31^ on black-and-white colobus and red-tailed guenon, the gut microbiota of Udzungwa red colobus includes only traces (<0.01%) of Fibrobacteres, Actinobacteria and TM7 phylotypes, while Verrucomicrobia, Spirochetes and Proteobacteria are well represented (Supplementary Table S2), although rarely described as components of the non-human primate gut.

**Figure 3:**
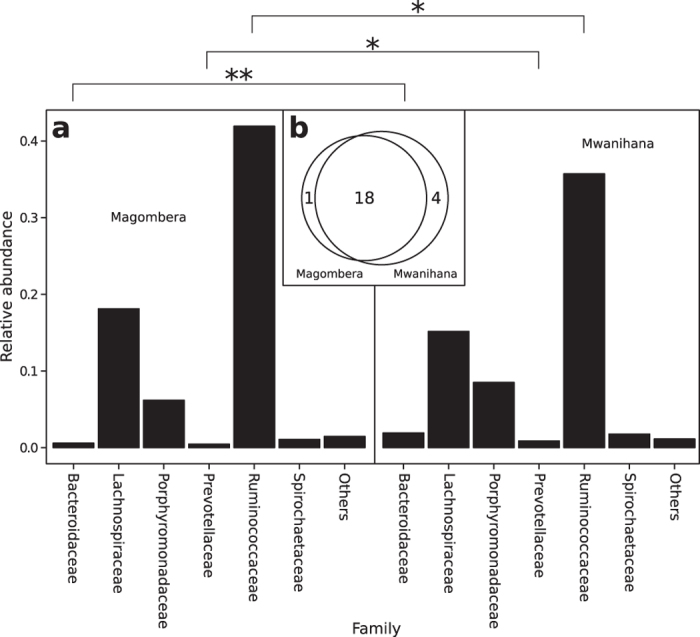
Relative abundance of bacterial families in faecal microbiota of Udzungwa red colobus social groups from the Magombera and Mwanihana forests. Relative abundance of Bacteroidaceae, Prevotellaceae and Ruminococcaceae were significantly different between social groups inhabiting the two forests (Welch two sample t-test, adjusted p-values: *p < 0.05; **p < 0.005) (**a**); Venn diagram shows unique and shared bacterial families in the Magombera and Mwanihana red colobus social groups (**b**). Singleton taxa were discarded.

The most represented families in the gut microbiota of red colobus from both forests included Ruminococcaceae, Lachnospiraceae, Porphyromonadaceae, Bacteroidaceae Spirochetaceae, Prevotellaceae, and Clostridiales_Incertae Sedis XIII (Fig. 3, Supplementary Table S2). As the Venn diagram in Fig. 3b illustrates, the Udzungwa red colobus gut microbiota includes 18 shared microbial families, as well as several unique ones: Clostridiaceae for Ma, and Verrucomicrobiaceae, Acetobacteraceae, Sphingomonadaceae and Enterobacteriaceae for Mw (Supplementary Table S2). The presence of acetogenic bacteria in Mw populations may be associated with the coexistence of specialist cellulolytic Ruminococcaceae species that produce formate, hydrogen and carbon dioxide that are then used by Acetobacteraceae to produce acetate^32^. The dominance of both Ruminococcaceae (genera *Oscillibacter*, *Ruminococcus*, *Pseudoflavonifractor* and *Faecalibacterium*) and Lachnospiraceae (genera *Roseburia*, *Coprococcus*, *Clostridium XIVa* and *XIVb*) likely reflects the folivorous diet of red colobus. These two bacterial families have high number glycoside hydrolase genes and specific metabolic pathways to cleave the cellulose and hemicellulose components of complex plant material and degrade a wide variety of polysaccharides^33^,^34^. Ruminococcaceae and Lachnospiracea, two of the most abundant families from the order Clostridiales found in the mammalian gut environment, are uniquely suited to degrade a wide variety of recalcitrant substrates such as those found in leaves, with identified key carbohydrate-active enzymes, sugar transport mechanisms, and metabolic pathways, suggesting that these two commensal bacterial families are specialists in degrading complex plant material^33^,^34^. In addition, Ruminococcaceae are enriched with carbohydrate-binding modules specialized in binding to cellulose, hemicellulose and xylan^35^.

While many of the microbial families are shared, there are some important differences in relative abundance of these taxa in red colobus populations from different forests. For example, Bacteroidaceae and Prevotellaceae were significantly more abundant in colobus from Mw (adjusted P < 0.005 and P < 0.05 respectively), while Ruminococcaceae and Clostridiales_Incertae Sedis XIII were more abundant in Ma individuals (adjusted P < 0.05, Fig. 3; Supplementary Table S2). This finding may relate to the different availability of food resources related to differences in plant diversity between Mw and Ma^27^,^29^,^30^ (Supplementary Table S3).

**Figure 4:**
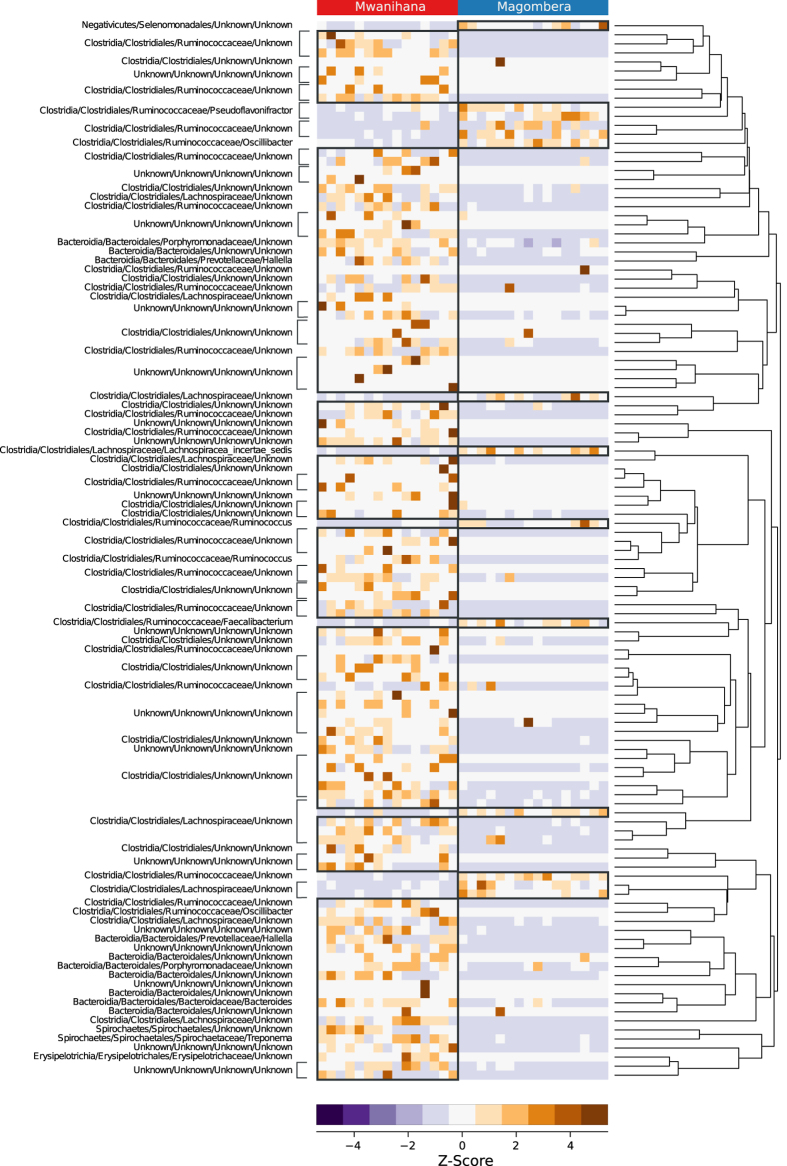
PhyloRelief analysis (Magombera vs. Mwanihana) guided by the Weighted UniFrac distance. Z-scores of the relative abundances are shown for significant OTUs (PhyloRelief selected clades with FDR-corrected P < 0.01, Kruskal-Wallis test). OTUs are classified in Phylum/Class/Order/Family/Genus format on the left side. Ultrametric pruned phylogenetic tree is shown on the right side.

We also found significant differences in the relative abundance of the genus *Faecalibacterium* (higher in Ma) and of *Halella* and *Bacteroides* (higher in Mw, P < 0.05, Supplementary Fig. S3). To define more precisely the taxa that are driving the differentiation of the microbiota of the two forests, we performed an analysis based on PhyloRelief^36^, a recent phylogenetic-based feature weighting algorithm for metagenomic data. The method unambiguously groups the relevant taxa into clades without relying on pre-defined taxonomic categories. The phylogenetic clades are weighted and ranked according to their abundance measuring their contribution to the differentiation of the classes of samples. The most relevant selected clades (Kruskal-Wallis test, P < 0.01) show that, beside these more evident differences, there is a general rearrangement of the taxa within the *Bacteroidales* and *Clostridiales* order, resulting in a lower diversity of the microbiota of the Ma individuals (Fig. 4).

Interestingly, all reads assigned to the Spirochaetaceae families are classified as *Treponema* genus (1.1% in Ma, 1.8% in Mw). Despite *Treponema* genus is a big genus with many different species, colonizing different animals, including very different ones such as the opportunistic pathogen *Treponema pallidum* and *Treponema denticola*, diverse species of *Treponema* are a typical component of the termite gut, as key players in lignin and xylan digestion^37^. Although highly speculative, termites are an occasional part of red colobus diet, especially during the rainy season^30^, thus this finding may suggest a possible “microbial horizontal transfer” of microorganisms present in the gut of the prey to the predator, possibly extending the range of ligneous substrates that can be digested. Moreover, termites are part also of the human diet in Africa, and *Treponema* has also been found in Burkina Faso children^11^ and more recently in hunter-gatherer and traditional agriculturalist communities in Peru^38^. It has been hypothesized^11^ that members of this genus could enhance the ability to extract calories from indigestible polysaccharides from a diet consisting mainly of cereals, legumes, and vegetables, as well as supplementing intake of animal protein, in fact in the Hazda population the increased *Treponema* among women may be an adaptation to the higher amount of plant fibre in their diet, especially from tubers^39^.

### Gut microbiota richness

**Figure 5:**
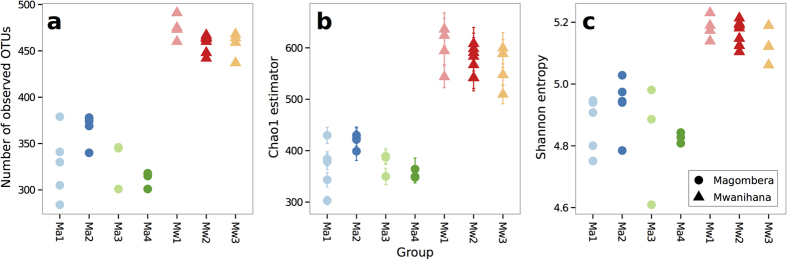
Measures of α-diversity for the gut microbiota of Udzungwa red colobus. For each individual, number of observed OTUs (**a**), the Chao1 estimator (**b**) and Shannon entropy (**c**) are given. Error bars in (**b**) indicate the Standard Error (SE). Symbols shapes and colours are coded according to Fig. 1c.

The complexity of microbiota has traditionally been measured using indices developed in theoretical ecology, such as the number of observed OTUs, the Chao1 estimator of species richness and Shannon entropy. All three of these indices indicate a consistent and significantly higher richness of gut microbiota in samples collected from Mw (Fig. 5). On average, observed bacterial richness was highest in the red colobus living in Mw with respect to subjects living in Ma (Fig. 5a, Wilcoxon rank-sum test, P = 2.3 × 10^−6^). Consistent with this observation, the Chao1 estimates of richness were substantially higher in all three Mw groups (Fig. 5b, P = 6.7 × 10^−9^) compared to the four Ma groups. In terms of Shannon diversity estimate the three groups living in Mw were consistently higher than the groups living in Ma (Fig. 5c, P = 6.7 × 10^−9^). Furthermore the number of operational taxonomic units (OTUs) per subject at 97% sequence similarity was higher in Mw than in Ma (mean per group 462 vs. 338 respectively), indicating that sampling site had significant effects on the richness of gut bacterial community composition as reported below.

The Udzungwa red colobus in continuous natural habitat, such as Mw, show a more diverse community of gut microbiota, in contrast to groups living in a forest fragment, presumably suboptimal habitat^27^,^28^, such as those in Ma. Such findings are in agreement with a similar pyrosequencing analysis of Mexican black howler monkeys occupying a variety habits from integral continuous evergreen forest, to degraded natural habitats, including captive conditions^21^. The authors demonstrate that non-human primate gut microbiota richness, diversity and composition vary strongly with habitat and that dietary shifts associated with habitat disturbance influence the gut microbiota composition^21^. In contrast, a recent study^22^ on Ugandan red colobus (*P. rufomitratus*) based on different fingerprint techniques (i.e. ARISA) on highly variable bacterial intergenic spacer region between 16S and 23S rRNA genes, did not reveal any alteration of gut microbial communities in fragmented forests. However, this study does not allow for deep resolution of the gut microbiota, and thus it is not directly comparable to our results.

### Gut microbiota profiles differ between forests

**Figure 6:**
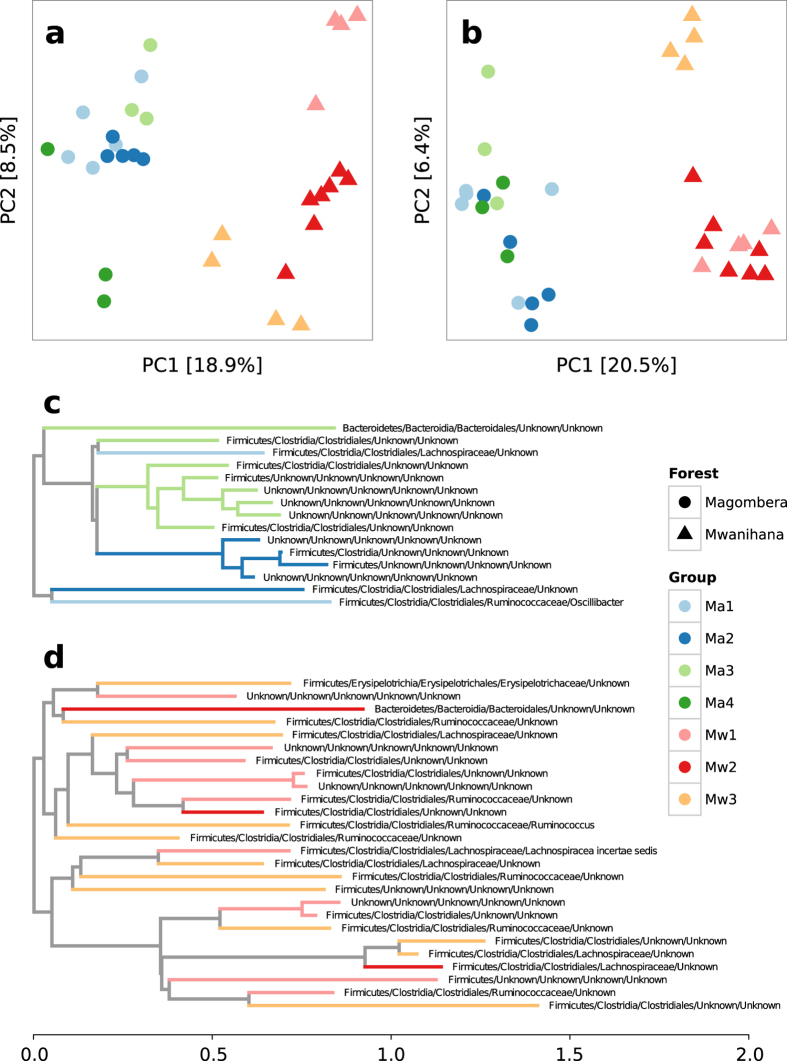
Between sample diversity and group specific clades. PCoA of the between samples distances measured using Bray-Curtis dissimilarity (**a**). PCoA of the between samples distances measured using unweighted UniFrac distance (**b**). Pruned phylogenetic trees with the clades driving differentiation of groups within the Ma (**c**) and Mw (**d**) forests (PhyloRelief weights ≥ 0.4). OTUs are classified in Phylum/Class/Order/Family/Genus format. Colours indicate in which group the clade is higher in terms of relative abundance. Symbols shapes and colours are coded according to Fig. 1c.

We inferred differences in microbiota profiles between samples using the Bray-Curtis and unweighted UniFrac^40^ dissimilarity indices, the latter taking into account the phylogenetic relationships between OTUs. A principal coordinates analysis (PCoA) analysis of the between-sample distance matrices is shown in Fig. 6 (PCoA using weighted UniFrac is shown in Supplementary Fig. S4). The first principal coordinate (PC) of the PCoA separates the two red colobus populations living in Mw and Ma forests (PERMANOVA P < 0.001 on Bray Curtis dissimilarity, Supplementary Table S4), while the second coordinate separates red colobus groups residing in different sampling sites within the same forest. Figure 6 shows that the gut microbiota of social groups within Ma are closely related; however, we found a clear segregation of Mw3 from the other two social groups within Mw. Mw1 and Mw2 are separated based on Bray-Curtis dissimilarity (Fig. 6a) but not by unweighted UniFrac (Fig. 6b). To define the OTUs that are driving the differentiation of the microbiota between the social groups within each forest more precisely, we performed two independent multi-class PhyloRelief analyses^36^ on Ma and Mw samples. In Fig. 6c,d the pruned phylogenetic trees (only clades with PhyloRelief weights ≥0.4 were selected) show how groups within forests are differentiated mainly by OTUs phylogenetically related to the class of Clostridia. This is just a taste of how much of the existing diversity can have previously uncharacterized functional implications in diverse microbiota and such differences show the current limit in knowledge and the need to in depth investigate these communities with finer functional studies.

### Allopatric and sympatric differentiation of gut microbiota

**Figure 7:**
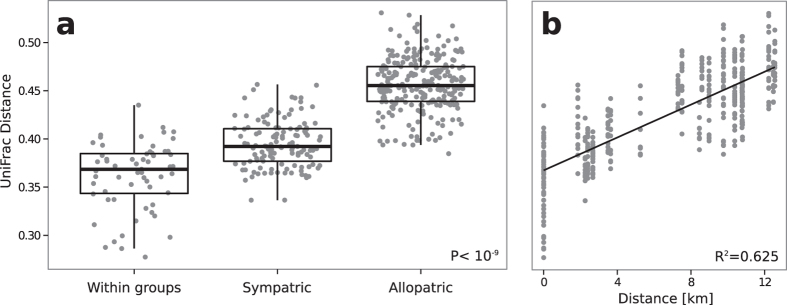
Microbiota profile distances as a function of physical distances. Pairwise unweighted UniFrac distances stratified by within social groups, and between sympatric and allopatric groups. P < 10^−9^ (Wilcoxon rank sum test, Bonferroni corrected) for all comparisons (**a**). Unweighted UniFrac distance plotted against geographical distances between GPS points of sample sites (**b**). The straight line is the linear least squares regression to the data.

In order to disentangle whether gut microbiota diversity is due to environmental or heritable factors we compare family social groups from sympatric (i.e. co-occurring in the same forest geographical location) and allopatric (i.e. geographically separated forest) host populations. Although geographical variation is often also confounded by variation in host genetic background, the population analysis of our study groups (using a set of 10 microsatellite loci) showed that gene flow between the two forests ceased recently (small but significant F_ST_). Therefore, only minor genetic differences between the colobus living in the Ma and Mw forests are expected (Ruiz-Lopez *et al.*, personal communication). Figure 7a shows that the median of the unweighted UniFrac distance between samples within the same social group is significantly smaller (P < 10^−9^, Wilkoxon rank sum test, Bonferroni correction) than that between individuals of sympatric groups (co-occurring in the same forest), which is in turn significantly smaller than the mean distance between individuals of allopatric groups (inhabiting different forests). Plausible explanations of this pattern include vertical transmission between parents and offspring, or horizontal transmission between group members that share the same home range and are in close contact with each other while feeding or allogrooming^41^. Similarly, in rural Papua New Guineans low variation (beta-diversity) among individuals was explained through environmental sharing and dispersal limitation^42^. However, we did not found significant differences between sympatric social groups within forests (Supplementary Fig. S5).

Due to the importance of diet in terms of presence/absence and relative abundance of bacterial phylotypes^2^,^4^,^11^,^12^, the overlap in dietary intake might also be an alternative explanation to the similarity in the gut microbial communities colonizing red colobus from the same social group.

Figure 7b shows that microbiota differentiation increases with increasing geographical distance between sampling sites, confirming previous results for wild vertebrate populations^17^,^18^,^19^,^20^. However, while differences found in the other studies were discovered over a large spatial distance, the differences reported in this study occur over a surprisingly small range from two to a maximum of 13 kilometers (Supplementary Table S5). Thus, environmental parameters (such as temperature, humidity or precipitation) are probably too similar to be potential causes for variation between sampling sites. Indeed, changes in diet associated with forest degradation^43^, increased access to human food, sugar cane and other sugar rich fruits in monkeys from Ma, as well as differences in the plant species found in the two forests (Supplementary Table S3) are more likely explanations for the signature of geographic distance, since differences in availability of food sources between the two forests have already been noted^27^,^29^,^30^,^43^.

### Metabolic functions associated to the gut microbiota profiles of red colobus

Co-evolution of beneficial microorganisms within the animal gastrointestinal tract fundamentally shapes animal physiology. Since gut microbiota may modulate the availability of ingested nutrients, such as fibre, and the consequent efficiency in energy-harvesting^11^,^44^,^45^, the metabolic potential of the gut microbiota is an important aspect to consider. Diet as well as genetic factors may influence gut microbiota, and thus host metabolic, hormonal and immune homeostasis. Bacterial species are known to carry and transfer operons containing genes for different metabolic functions. Different bacterial species are enriched for certain functions and these correlations have been categorized in well-organized databases^46^. Therefore, in order to clarify how phylogenetic differences between the gut microbiota of individuals living in the two forests impact their metabolic potential, we applied PICRUSt (Phylogenetic Investigation of Communities by Reconstruction of Unobserved States), a computational approach used to predict the functional composition of a metagenome using marker gene data and a database of reference genomes^47^. PICRUSt implements an extended ancestral-state reconstruction algorithm to predict which gene families are present and then combines gene families to estimate the composite metagenome. In order to test prediction accuracy of PICRUSt, the authors applied the computational method to diverse metagenomic data sets such as humans, soils, other mammalian guts and the Guerrero Negro, a hypersaline microbial mat, showing that the phylogenetic information contained in 16S marker gene sequences is sufficiently well correlated with genomic content to yield accurate predictions when related reference genomes are available. Although the limitations of this approach must be considered, related to reference genome sequence databases, we use PICRUSt as metagenome inference method on our 16S rDNA dataset. The analysis performed on samples from each forest reveals functional classes (KEGG categories) with a remarkably similar assignment, regarding carbohydrate metabolism, energy metabolism, fatty acid metabolism, glycan biosynthesis and metabolism, lipid metabolism, aminoacid metabolism, protein digestion, absorption and export, identifying a core of metabolic capacities shared between the two populations of animals (Supplementary Fig. S6 and Supplementary Table S6). However, within the main KEGG categories there are also major and significant differences, attributable to the different environments of the two forests and probably to different food source availability. The plant polysaccharides commonly consumed by mammals are rich in xylan, pectin, and cellulose. The fibre-rich diet of colobus monkeys has resulted in a gut microbiome enriched in metabolic functions such as carbohydrate metabolism, glycosaminoglycan degradation and glycosyltransferases, essential for glycan biosynthesis and metabolism (Supplementary Fig. S6), similar to that of other herbivorous mammals^48^ (e.g. ruminants) than to other primates^2^.

The fact that more fructose and mannose metabolism is associated with the Mw microbiome (Supplementary Fig. S6) could be related to a lower number of Ruminococcaceae (Fig. 3 and Supplementary Table S2), suggesting that the red colobus living in this forest may have a more varied diet, including more fruit.

**Figure 8:**
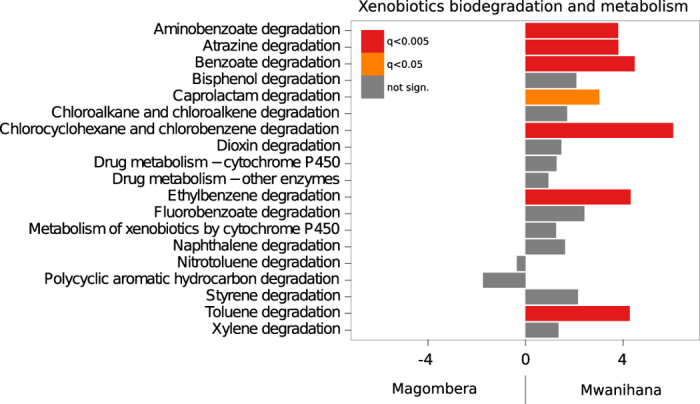
T-test statistics of the relative abundances of KEGG modules grouped by biochemical pathway. Colours of the bars indicate significance measured by False Discovery Rate.

Importantly all the microbial species known to be involved in the xenobiotic degradation pathway (caprolactam degradation, aminobenzoate degradation, atrazine degradation, benzoate degradation, chlorocyclohexane and chlorobenzene degradation, ethylbenzene degradation, toluene degradation) are significantly over-represented in samples from Mw, together with all the functional genes and categories needed for this complex degradation pathway (Fig. 8). This differential enrichment in functions is in agreement with the presence of plant species in Mw rich in potentially harmful tannins (Ref. 30Tables 47–51, pages 303–305; i.e., *Antiaris toxicaria welwitschii* is among the most frequently eaten plant species). This suggests that the microbial community involved in detoxification of potentially toxic compounds present in Mw red colobus has been lost in Ma, where, despite the small geographical distance, these plant species are no longer present (ref. 30Tables 47–51, pages 303–305). Taken together the functional implications of the differences in the microbiota composition from colobus from Ma and Mw likely stem from differences in the dietary plant species present in the two forests.

The variability of the microbial community is counterbalanced by the robustness of the functional composition. Microbiota profiles of red colobus living in the two different forests studied share a common set of microorganisms and microbial functions, underpinning a core of metabolic capacities for folivorous primates. All major types of microorganisms, including proteolytic bacteria and cellulolytic species, as well as some niche specialists, were present. Yet the abundance analyses showed a prevalence of Ruminococcaceae and Lachnospiraceae, families that are known to be highly specialized in the degradation of complex plant material^49^. These compounds are then fermented and converted into short chain fatty acids (mainly acetate, butyrate, and propionate) that can be absorbed and used for energy by the host. This finding is corroborated by functional analyses, showing a core enriched in genes involved in extracting energy from fibres, in addition to fat, sugar and protein metabolism. All functional classes in common between the two forest groups had a remarkably similar assignment, suggesting that gut microbial communities of red colobus, living in different environments, maintain a stable set of functions and metabolic potential, even when their composition in terms of taxa fluctuated.

Despite the core represented by some metabolic capacities in both forests, we found certain metabolic functions to be enriched in Mw. The Mw gut microbiota possessed the relevant metabolic potential to detoxify xenobiotics present in the folivourous diet. These detoxifying pathways have apparently been lost in Ma red colobus, possibly as a consequence of environmental degradation and loss of plant diversity.

## Conclusions

Overall, we show that microbiota richness and diversity are reduced in Ma, likely as result of dietary changes enforced by human disturbance and habitat degradation. Therefore, the changes in the taxonomic and functional pattern of gut microbiota species composition of free-living primates could be used as an indicator of habitat degradation and fragmentation. Our results also indicate how conservation interventions must also be adjusted to protect natural diversity at all levels.

The results also propose a mechanistic explanation of how subtle alterations in the environment, restricting or altering the availability of food resources, could influence the conservation of endangered species. A large fraction of the Udzungwa red colobus microorganisms have not been described as part of the human or primate gut microbiome thus far and are likely to have an environmental origin, possibly limited in time to the passage through the host gut, being therefore passengers rather than colonizers, or conditional colonizers that can be easily lost because of subtle changes in diet.

Future studies should examine the relationship between red colobus gut microbiota composition and the environment to support the environmental origin of microorganisms, and the relationship with health status, by measuring factors that are likely be influenced by microbiome homeostasis such as immune response, and parasite load and diversity. Finally, a careful assessment of the reproductive fitness of social groups could help disentangle the environmental pressures determining gut microbiota community structure and function, and how the resulting changes to gastrointestinal microbial communities in turn, impact on metabolism efficiencies and fitness of the hosts. Overall, we highlight the relevance of microbial ecology investigations to assess potential effects of diet components in wild primates living in natural versus human-modified habitats. Thus, our results encourage deeper investigations on wild populations inhabiting ecologically relevant but severely exploited areas, which deserve specific conservation strategies to protect the environment itself and more importantly their wider ecosystem.

## Methods

### Study animals

Udzungwa red colobus are forest canopy-dwellers living in large social groups (up to 80 individuals), selective feeders with a predominantly folivorous diet, although insects are occasionally eaten, and foregut fermenters with a complex four-chambered stomach^50^. The stomach is enlarged, allowing for food accumulation and longer digestion. As in ruminants, such long digestive retention time may enable host bacteria to ferment the dietary polysaccharides and provide the host with dietary energy to survive on a herbivorous diet. Recent ecological studies of several populations found that abundance and demographic parameters are affected by forest size, human pressure and habitat integrity^28^,^43^.

### Study site

The Udzungwa Mountains (Udzungwas hereafter) extend over 19 000 km^2^ and are divided into large forest blocks by a combination of natural factors (e.g. geology, climate, terrain morphology) and smaller ones as a result of agricultural activities^28^. We sampled stools from seven social groups from two populations inhabiting Mwanihana (Mw; part of a large forest block) and Magombera (Ma; a fragment) separated by a minimum of 6 kilometers of unsuitable habitat (cultivated fields) since 1950^27^ (Fig. 1).

### Faecal sampling

Faecal samples were collected during two dry seasons (January-February and August-September 2013), similar in food availability and individual activity pattern^30^. Social groups moving through the canopy were followed unobtrusively from the ground, and stools were collected once from each social group during a single defecation event (when several individuals defecate simultaneously on the forest floor). We collected fifteen samples from three Mw social groups, and 16 samples from four Ma groups (mean: 4.4 samples per group; range: 3–7; Fig. 1). Although freezing is considered to be the best method for long term storage of bacterial DNA from faeces, this method cannot be usually applied for samples of wild primates collected in the challenging condition of the tropical forest. In order to find an alternative conservation method more efficient respect to ethanol, we used RNAlater® Stabilization Solution as described by Vlkova *et al.*^51^ and Larsen *et al.*^52^. We stored 0.5 g of each feces (representing one individual) in a 15 ml polypropylene tube pre-filled with 5 ml of RNAlater® Stabilization Solution (Ambion, Life Technologies, Monza, Italy), stored at ambient temperature for up to 8 days, shipped to Italy, and kept at −20 °C until DNA extraction. Samples were collected without direct contact or interaction with the animals and under permit approval from the Tanzania Commission for Science and Technology (COSTECH), Tanzania Wildlife Research Institute (TAWIRI) and Tanzania National Parks (TANAPA). Our data collection procedure adhered to the legal requirements and complied with the laws governing wildlife research in Tanzania.

### DNA Extraction, Amplicons construction of 16S rRNA Gene, Library construction and pyrosequencing

Genomic DNA was extracted from each sample using the QIAamp DNA Stool Kit (Qiagen, Milano, Italy) following the manufacturers’ instructions, quality-assessed by gel electrophoresis and the NanoDrop spectrophotometer (Thermo Fisher, Waltham, Ma).

For each sample, we amplified the 16S rRNA gene using the special fusion primer set specific for V1-V3 hypervariable regions (27-Forward: 5′-AGAGTTTGATCMTGGCTCAG-3′^53^ and 533-Reverse: 5′-TTACCGCGGCTGCTGGCAC-3′^54^), using FastStart High Fidelity PCR system (Roche Life Science, Milano, Italy) (Details in *SI* Materials and Methods). The PCR products (three replicates) of the 31 samples (16 relative to Ma forest, 15 to Mw forest) were analyzed by gel electrophoresis and cleaned using the AMPure XP beads kit (Beckman Coulter, Brea, CA, USA) following the manufacturer’s instructions, quantified via quantitative PCR using the Library quantification kit Roche 454 titanium (KAPA Biosystems, Boston, Ma) and pooled in equimolar proportion in a final amplicon library. The 454 pyrosequencing was carried out on the GS FLX+ system using the XL+ chemistry following the manufacture’s recommendations.

### Data analysis

Pyrosequencing resulted in a total of 344,938 16S rDNA reads with a mean of 11,127 sequences per sample. Average sequence lengths were 511 nt (±SD 34) and 513 nt (±SD32) for the first and second run, respectively. Raw 454 files were demultiplexed using the Roche’s sff file software, and available at the European Nucleotide Archive (www.ebi.ac.uk) under the accession study PRJEB8977. Sample accessions and metadata are available in Supplementary Table S7. Reads were preprocessed using the MICCA pipeline^55^ (version 0.1, http://compmetagen.github.io/micca/). Forward and reverse primer trimming and quality filtering were performed using micca-preproc (parameters -f AGAGTTTGATCMTGGCTCAG -r GTGCCAGCAGCCGCGGTAA -O 15 -l 300 -q 22) truncating reads shorter than 300 nt. De-novo sequence clustering, chimera filtering and taxonomy assignment were performed by micca-otu-denovo (parameters -s 0.97 -c): operational taxonomic units (OTUs) were assigned by clustering the sequences with a threshold of 97% pair-wise identity, and their representative sequences were classified using the RDP^56^ software version 2.7. Template-guided multiple sequence alignment (MSA) was performed using PyNAST^57^ (version 0.1) against the multiple alignment of the Greengenes database^58^ (release 13_05) filtered at 97% similarity. Finally, a phylogenetic tree was inferred using FastTree^59^ and micca-phylogeny (parameters: -a template —template-min-perc 50). Sampling heterogeneity was reduced by rarefaction (4067 sequences per sample). Alpha (within-sample richness) and beta-diversity (between-sample dissimilarity) estimates were computed using the phyloseq R package^60^. Permutational MANOVA (PERMANOVA) statistical tests were performed using the R package vegan (adonis() function) with 999 permutations. To compare the relative abundances of OTUs between the two forests, two-sided, unpaired Welch t-statistics were computed using the function mt() in the phyloseq package and the p-values were adjusted for multiple comparison controlling the family-wise Type I error rate (minP procedure^61^). In order to clarify how phylogenetic differences between the gut microbiota of individuals living in the two forests impact their microbial metabolic potential, we applied PICRUSt^47^ (Phylogenetic Investigation of Communities by Reconstruction of Unobserved States). PICRUSt uses an extended ancestral-state reconstruction algorithm to predict which gene families are present and then combines gene families to estimate the composite metagenome starting from the taxonomic composition estimated from 16S rDNA data. Starting from a table of OTUs with associated Greengenes identifiers, we obtained the final output from metagenome prediction as an annotated table of predicted gene family counts for each sample, where the encoded function of each gene family be orthologous groups or other identifiers such as KEGG orthologs (KOs).

## Additional Information

**How to cite this article**: Barelli, C. *et al.* Habitat fragmentation is associated to gut microbiota diversity of an endangered primate: implications for conservation. *Sci. Rep.* **5**, 14862; doi: 10.1038/srep14862 (2015).

## Supplementary Material

Supplementary Material

Supplementary Table S6

Supplementary Table S7
