# Supplementary material for: Habitat fragmentation is associated to gut microbiota diversity of an endangered primate: implications for conservation
Source: Sci Rep. 2015 Oct 7;5:14862. doi: 10.1038/srep14862 (PMC4595646; doi:10.1038/srep14862)
Supplement: Supplementary Material [file srep14862-s1.pdf]

Habitat fragmentation is associated to gut microbiota diversity of an endangered primate: implications for conservation

Supplementary Information

CLAUDIA BARELLI[1,2,3*], DAVIDE ALBANESE[4*], CLAUDIO DONATI[4], MASSIMO PINDO[5], CHIARA DALLAGO[5], FRANCESCO ROVERO[2], DUCCIO CAVALIERI[4], KIERAN MICHAEL TUOHY[6], HEIDI CHRISTINE HAUFFE[1] & CARLOTTA DE FILIPPO[6,7]

[1] Department of Biodiversity and Molecular Ecology, Research and Innovation Centre, Fondazione Edmund Mach, Via E. Mach 1, 38010 S. Michele all'Adige, Trento, Italy

[2] Tropical Biodiversity Section, MUSE – Museo delle Scienze, Corso del Lavoro e della Scienza 3, 38123 Trento, Italy

[3] Reproductive Biology Unit, German Primate Centre, Leibniz Institute for Primate Research, Kellnerweg 4, 37077 Göttingen, Germany

[4] Department of Computational Biology, Research and Innovation Centre, Fondazione Edmund Mach, Via E. Mach 1, 38010 S. Michele all'Adige, Trento, Italy

[5] Department of Genomics and Biology of Fruit Crops, Research and Innovation Centre, Fondazione Edmund Mach, Via E. Mach 1, 38010 S. Michele all'Adige, Trento, Italy

[6] Department of Food Quality and Nutrition, Research and Innovation Centre, Fondazione Edmund Mach, Via E. Mach 1, 38010 S. Michele all'Adige, Trento, Italy

[7] Department of Neurosciences, Psychology, Drug Research and Child Health, University of Florence, Florence, Italy

[*] These authors contributed equally to this work.

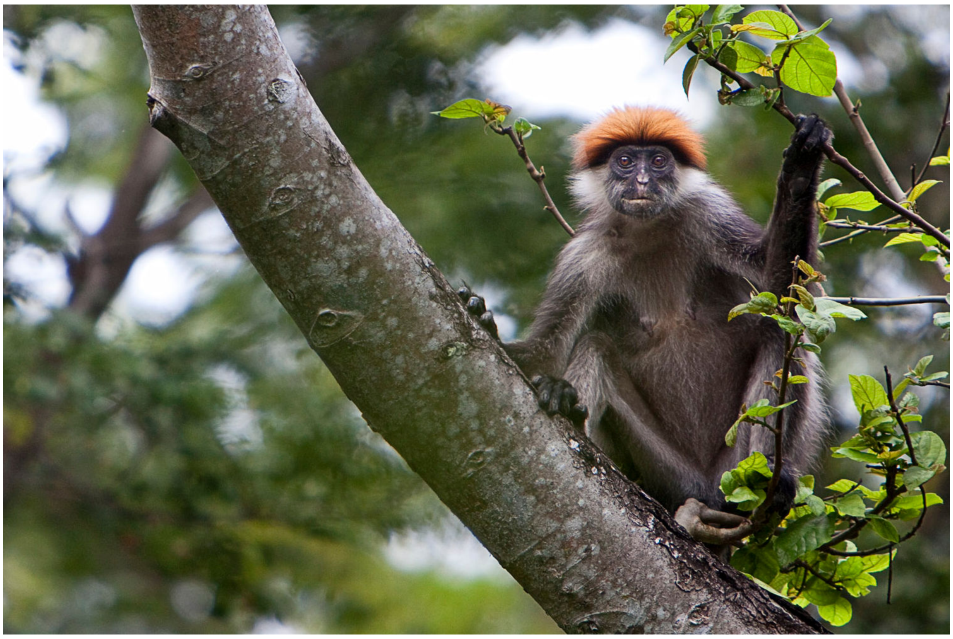

Photo courtesy of Raffaele Merler.

Supplementary Figure S1. Red colobus monkeys (*Procolobus gordonorum*) living in the Udzungwa Mountains (Tanzania) are arboreal forest dwellers. This primate species provides an excellent model for investigating variation in gut microbial communities across host habitats because it has a specialized diet, making it particularly sensitive to habitat degradation. Colobines are mainly folivorous, selective feeders, preferentially consuming fresh young shoots and leaves[1] which typically have a higher energy and nutrient concentration and lower fibre content than mature leaves[2]. Moreover, like other colobines, the Udzungwa red colobus is a foregut fermenter with a complex four-chambered stomach[3]. The stomach is also enlarged, allowing for food accumulation and prolonged digestion. As in ruminants, such a protracted digestive retention time enables intestinal bacterial communities to ferment dietary polysaccharides, allowing the host to extract sufficient energy from its herbivorous diet.

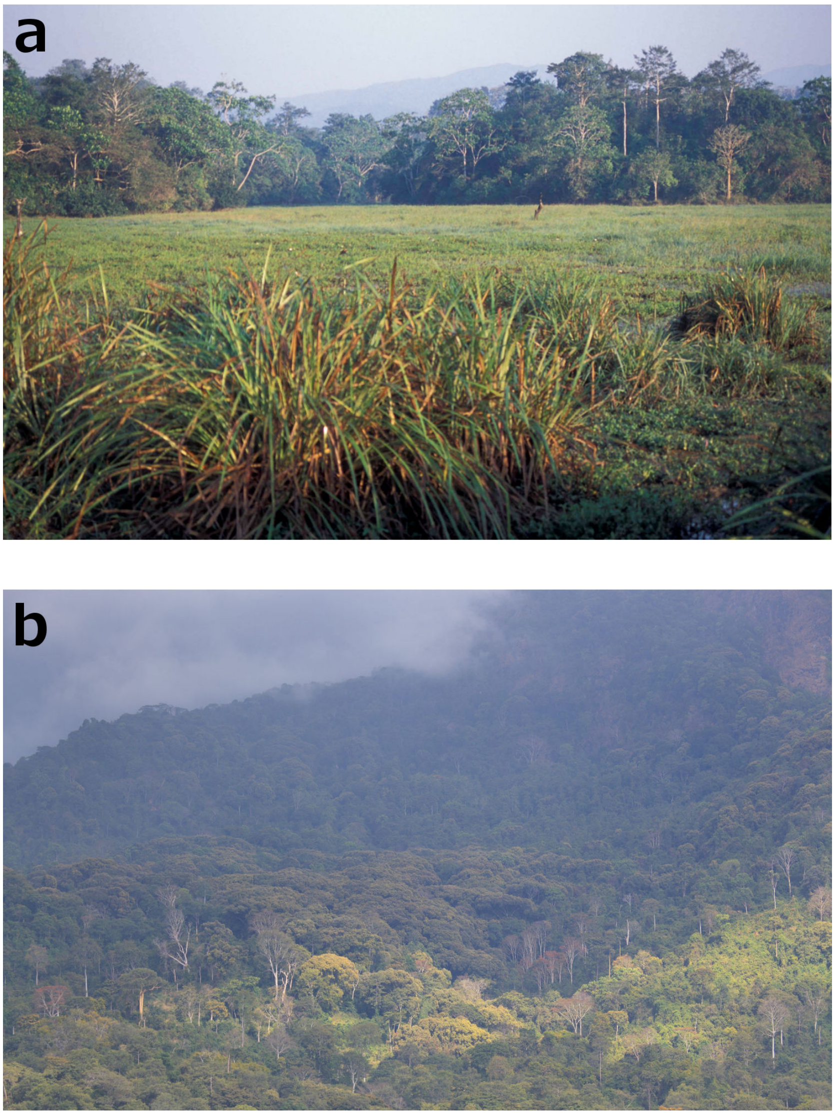

Photo courtesy of Francesco Rovero (**a**) and Rasmus Gren Havmøller (**b**).

Supplementary Figure S2. Ground views of the two selected forest blocks in the Udzungwa Mountains, Magombera (**a**) and Mwanihana (**b**). The Udzungwa Mountains (7°40′ S to 8°40′ S and 35°10′ E to 36°50′ E; Fig. 1, main text) extend over 19,000 km^2 and represent a mosaic of moist forest blocks of variable sizes (from 12 km^2 to >500 km^2), interspersed with naturally drier areas and/or habitat modified by agriculture, human settlements, and logging[4]. Rainfall in the moister forest slopes ranges from 2,000 to 2,500 mm/yr, concentrated in two periods: November–December and March–May. Overall, elevation spans from 270 (Kilombero Valley in the eastern side) to 2,576 m a.s.l. (Mount Luhomero). The two study forests have contrasting habitat types, elevation gradients, and protection levels: Magombera (Ma) is a remnant, ground-water and evergreen lowland forest patch which is separated from other forest blocks by the surrounding intensive agriculture and human settlements[5]. In addition, Ma is one of the smallest forest patches in the Udzungwas (Fig. 1 main text), it is not protected and 40% is heavily degraded[5], which make the forest encroached by nearby villagers for firewood, pole and timber harvesting and occasionally for hunting. Instead, the Mwanihana forest (Mw) is a forest escarpment with forest zones ranging from lowland deciduous to montane evergreen[6]. Mw is well protected and it is part of the Udzungwa Mountains National Park (UMNP).

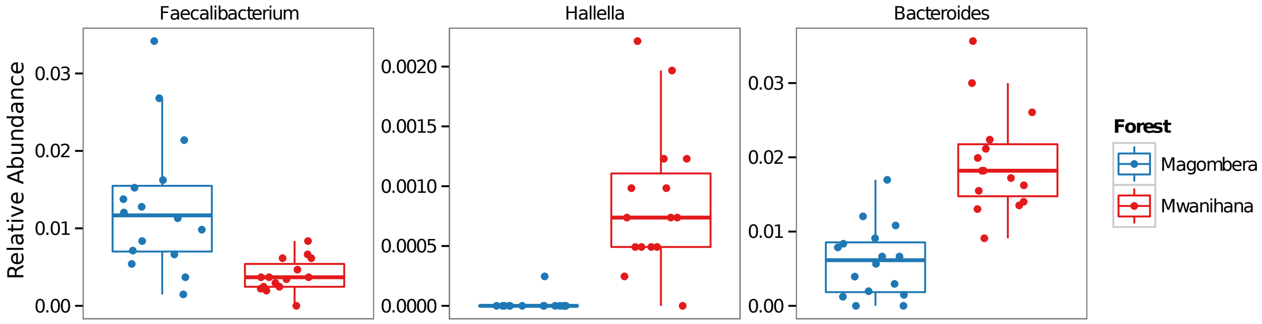

Supplementary Figure S3. Box-plots of significant bacterial population differences at the Genus level (Welch's t-test, P<0.05).

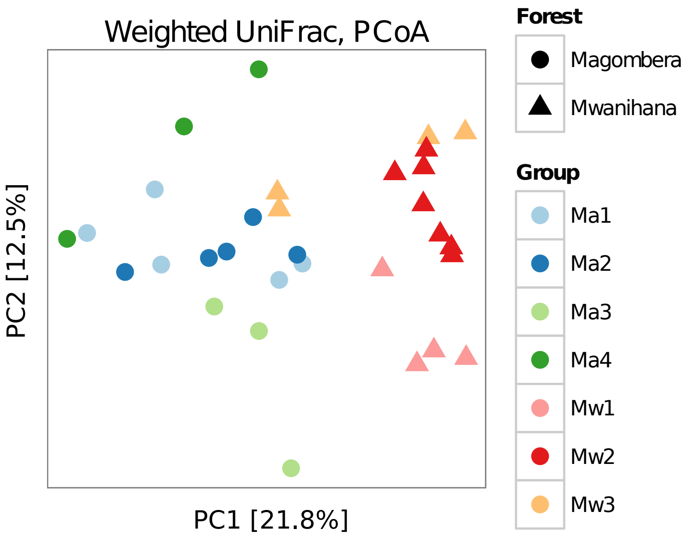

Supplementary Figure S4. PCoA of the between samples distances measured using weighted UniFrac distance.

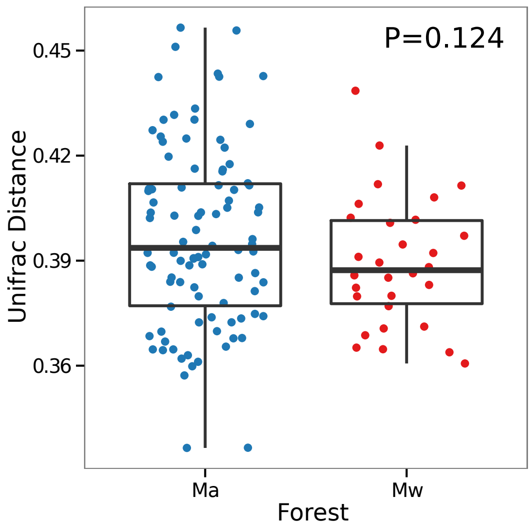

Supplementary Figure S5. Pairwise unweighted UniFrac distances between sympatric groups, stratified by forest (P=0.124, Wilcoxon rank sum test).

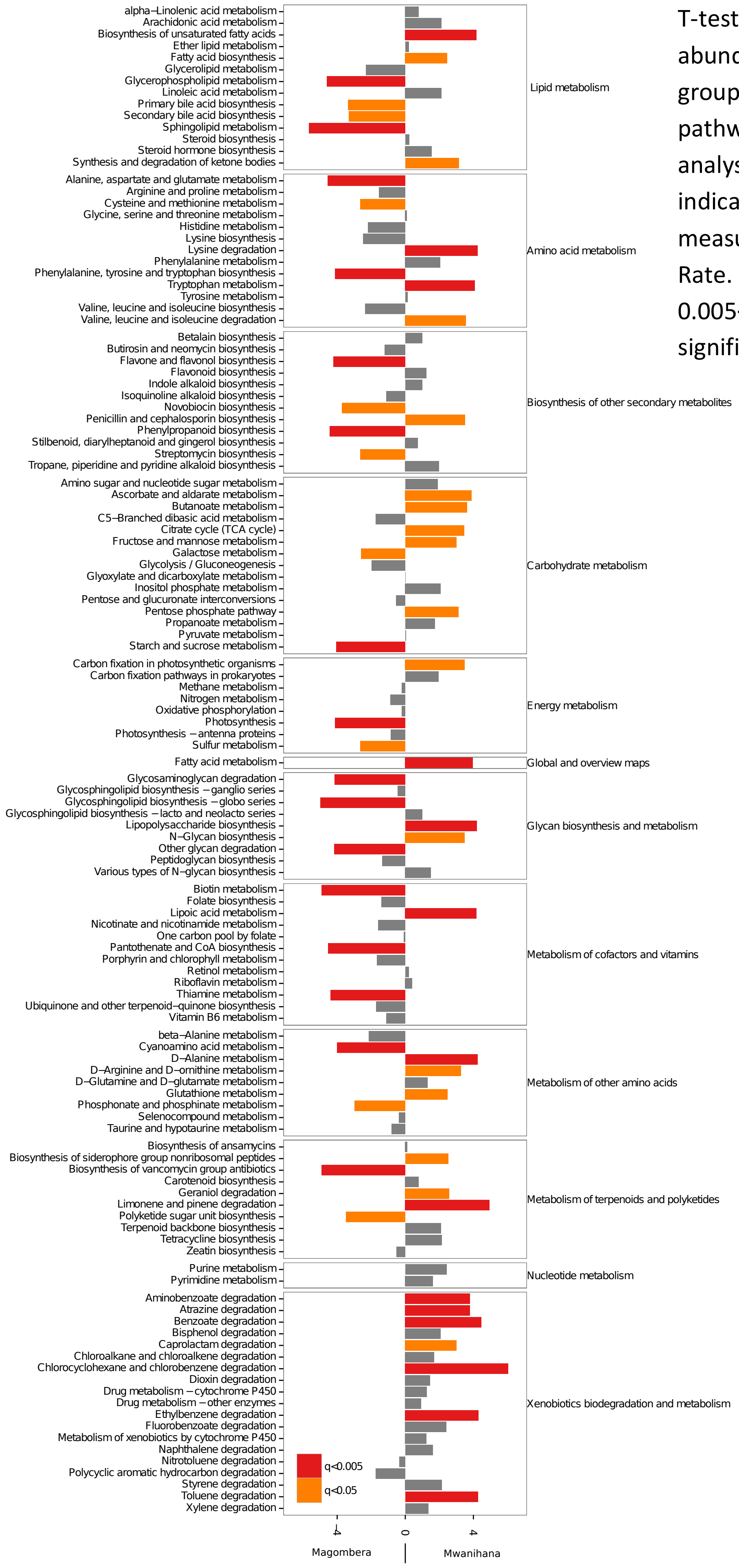

Supplementary Figure S6. T-test statistics of the relative abundances of KEGG modules grouped by biochemical pathway relative to PICRUSt analysis. Colours of the bars indicate significance measured by False Discovery Rate. Red: q<0.005; orange: 0.005<=q<0.05; grey: not significant.

ID	Altitude	Group	Forest	Lat	Long	Date
**MA03_01**	282	Ma1	Magombera	-7.8127	36.975	31/01/13
**MA03_02**	282	Ma1	Magombera	-7.8127	36.975	31/01/13
**MA03_03**	282	Ma1	Magombera	-7.8127	36.975	31/01/13
**MA03_04**	282	Ma1	Magombera	-7.8127	36.975	31/01/13
**MA03_05**	282	Ma1	Magombera	-7.8127	36.975	31/01/13
**MA06_01**	283	Ma2	Magombera	-7.8335	36.9811	30/01/13
**MA06_02**	283	Ma2	Magombera	-7.8335	36.9811	30/01/13
**MA06_03**	283	Ma2	Magombera	-7.8335	36.9811	30/01/13
**MA06_04**	283	Ma2	Magombera	-7.8335	36.9811	30/01/13
**MA06_05**	283	Ma2	Magombera	-7.8335	36.9811	30/01/13
**MA20_01**	264	Ma3	Magombera	-7.83665	36.99762	01/02/13
**MA20_03**	264	Ma3	Magombera	-7.83665	36.99762	01/02/13
**MA20_04**	264	Ma3	Magombera	-7.83665	36.99762	01/02/13
**MA25_01**	283	Ma4	Magombera	-7.81587	36.95482	31/01/13
**MA25_02**	283	Ma4	Magombera	-7.81587	36.95482	31/01/13
**MA25_04**	283	Ma4	Magombera	-7.81587	36.95482	31/01/13
**Mw_01**	492	Mw1	Mwanihana	-7.849439	36.884545	28/08/13
**Mw_02**	492	Mw1	Mwanihana	-7.849439	36.884545	28/08/13
**Mw_09**	492	Mw1	Mwanihana	-7.849439	36.884545	28/08/13
**Mw_12**	492	Mw1	Mwanihana	-7.849439	36.884545	28/08/13
**Mw_07**	643	Mw2	Mwanihana	-7.825414	36.887414	30/08/13
**Mw_08**	643	Mw2	Mwanihana	-7.825414	36.887414	30/08/13
**Mw_10**	643	Mw2	Mwanihana	-7.825414	36.887414	30/08/13
**Mw_11**	643	Mw2	Mwanihana	-7.825414	36.887414	30/08/13
**Mw_13**	643	Mw2	Mwanihana	-7.825414	36.887414	30/08/13
**Mw_14**	643	Mw2	Mwanihana	-7.825414	36.887414	30/08/13
**Mw_15**	643	Mw2	Mwanihana	-7.825414	36.887414	30/08/13
**Mw_03**	572	Mw3	Mwanihana	-7.77507	36.902821	01/09/13
**Mw_04**	572	Mw3	Mwanihana	-7.77507	36.902821	01/09/13
**Mw_05**	572	Mw3	Mwanihana	-7.77507	36.902821	01/09/13
**Mw_06**	572	Mw3	Mwanihana	-7.77507	36.902821	01/09/13

Supplementary Table S1. Sample metadata. Information includes sample IDs, site altitude, animal group, forest, latitude, longitude, date of sampling.

Phylum		
	Magombera	Mwanihana
Firmicutes	75.416	65.977
Bacteroidetes	12.117	17.543
Unknown	9.145	11.679
Verrucomicrobia	1.697	1.954
Spirochaetes	1.088	2.008
Proteobacteria	0.426	0.734
Tenericutes	0.077	0.075
Elusimicrobia	0.022	0.010
TM7	0.005	0.008
Cyanobacteria/Chloroplast	0.003	0.008
Actinobacteria	0.003	0.002
Fibrobacteres	0.002	0.000
Lentisphaerae	0.000	0.002

Family		
	Magombera	Mwanihana
Ruminococcaceae	41.932	35.731
Unknown	30.133	34.861
Lachnospiraceae	18.115	15.171
Porphyromonadaceae	6.195	8.514
Spirochaetaceae	1.088	1.769
Clostridiales_Incertae Sedis XIII	0.718	0.416
Bacteroidaceae	0.598	1.934
Prevotellaceae	0.467	0.882
Erysipelotrichaceae	0.330	0.277
Campylobacteraceae	0.092	0.092
Anaeroplasmataceae	0.077	0.075
Peptococcaceae 1	0.065	0.025
Clostridiaceae 1	0.063	0.000
Sutterellaceae	0.058	0.095
Elusimicrobiaceae	0.022	0.010
Oxalobacteraceae	0.020	0.051
Bacillaceae 1	0.014	0.007
TM7_genera_incertae_sedis	0.005	0.008
Chloroplast	0.003	0.008
Mycobacteriaceae	0.002	0.000
Streptomycetaceae	0.002	0.000
Peptostreptococcaceae	0.002	0.023
Fibrobacteraceae	0.002	0.000
Leuconostocaceae	0.000	0.002
Verrucomicrobiaceae	0.000	0.003
Acetobacteraceae	0.000	0.015
Sphingomonadaceae	0.000	0.008
Pseudomonadaceae	0.000	0.002
Victivallaceae	0.000	0.002

Supplementary Table S2. Mean relative abundance (%) of OTUs at the phylum and family level in the Magombera and Mwanihana forests.

Mwanihana	Magombera
Agelaea unknown	*Allophylus pervillei*
Alangium chinense	*Anthocleista grandiflora*
Albizia gummifera	*Aoranthe penduliflora*
Allanblackia ulugurensis	*Burttdavya nyasica*
Alsodeiopsis schumannii	*Calycosiphonia spathicalyx*
Anthocleista grandiflora	*Cordia peteri*
Aphloia theiformis	*Craterispermum schweinfurthii*
Baphia unknown	*Dialium holtzii*
Beilschmiedia kweo	*Didymosalpinx norae*
Bersama abyssinica	*Diospyros abyssinica*
Bridelia micrantha	*Diospyros mespiliformis*
Canthium oligocarpum	*Diospyros zombensis*
Canthium unknown	*Dracaena mannii*
Casearia gladiiformis	*Erythrophleum suaveolens*
Cassipourea gummiflua	*Eugenia capensis*
Cassipourea malosana	*Gardenia posoquerioides*
Cassipourea pachysiphon	*Guibourtia schliebenii*
Cephalosphaera usambarensis	*Haplocoelopsis africana*
Chionanthus mildbraedii	*Isoberlinia scheffleri*
Chrysophyllum gorungosanum	*Khaya anthotheca*
Chrysophyllum polystachyus	*Kraussia speciosa*
Cleistanthus polystachyus	*Leptactina platyphylla*
Coffea mongensis	*Lettowianthus stellatus*
Cola greenwayi	*Ochna holstii*
Cola unknown	*Oxyanthus pyriformis*
Combretum unknown	*Oxyanthus pyriformis subsp. tanganyikensis*
Craterispermum schweinfurthii	*Parkia filicoidea*
Croton sylvaticus	*Polyalthia verdcourtii*
Dialium holtzii	*Pseudobersama mossambicensis*
Dichapetalum unknown	*Psychotria schliebenii*
Didymosalpinx norae	*Pterocarpus mildebraedii*
Diospyros amaniensi	*Rawsonia lucida*
Diospyros amaniensis	*Rinorea ferruginea*
Dovyalis unknown	*Rothmannia macrosiphon*
Dracaena mannii	*Sorindeia madagascariensis*
Drypetes gerrardii	*Synsepalum brevipes*
Drypetes mannii	*Tabernaemontana pachysiphon*
Drypetes parvifolia	*Tapura fischeri*
Drypetes usambarensis	*Tarenna pavettoides*
Drypetes usambarica	*Tetrapleura tetraptera*
Englerophytum natalense	*Treculia africana*
Ficalhoa laurifolia	*Vepris amaniensis*
Ficalhoa laurifolia Hiern	*Vitex doniana*
Ficus unknown	*Vitex mossambicensis*
Flacourtia indica	*Xylopia longipetala*
Funtumia africana	
Garcinia buchananii	
Garcinia kingaensis	
Grewia mildbraedii	

Halleria lucida
Harungana madagascariensis
Heinsenia diervilleoides
Hirtella zanzibarica
Ilex mitis
Ixora scheffleri
Keetia unknown
Kraussia speciosa
Lagynias pallidiflora
Lagynias rufescens
Lepidotrichilia volkensii
Leptonychia usambarensis
Lettowianthus stellatus
Macaranga capensis
Memecylon unknown
Milicia excelsa
Mnunganunga unknown
Monodora unknown
Morella salicifolia
Myrianthus holstii
Newtonia buchananii
Ochna holstii
Ochna macrocalyx
Ocotea kenyensis
Octoknema orientalis
Oncoba welwitschii
Oxyanthus speciosus
Parinari excelsa
Parkia filicoidea
Pauridiantha paucinervis
Phoenix reclinata
Placodiscus amaniensis
Polyalthia suaveolens
Pteleopsis myrtifolia
Pterocarpus tinctorius
Pycnocoma littoralis
Quassia undulata
Rapanea melanophloeos
Rawsonia lucida
Rawsonia macrosiphon
Rawsonia rufescens
Ricinodendron heudelotii
Rothmannia macrosiphon
Rytigynia pseudolongicaudata
Shirakiopsis elliptica
Sorindeia madagascariensis
Strombosia scheffleri
Strychnos mellodora
Synsepalum cerasiferum
Synsepalum kaessneri

Syzygium guineense
Tabernaemontana pachysiphon
Tabernaemontana pallidiflora
Tabernaemontana ventricosa
Tarenna pavettoides
Tarenna unknown
Tiliacora funifera
Treculia africana
Trema orientalis
Tricalysia coriacea
Tricalysia pallens
Tricalysia unknown
Trichilia dregeana
Trilepisium madagascariense
Turraea unknown
Uncaria africana
Uvariodendron pycnophyllum
Vepris amaniensis
Vepris simplicifolia
Vernonia unknown
Xymalos monospora
Zanthoxylum gilletii

Supplementary Table S3. Plant species found in the two forests, Mwanihana (data from [7]) and Magombera (data from [5]).

	Metric	F	R^2	P
**Between Forests**	u. UniFrac	6.985	0.194	0.001
	w. UniFrac	5.983	0.171	0.001
	Bray-Curtis	6.113	0.174	0.001
**Between groups (Magombera)**	u. UniFrac	1.658	0.293	0.001
	w. UniFrac	1.982	0.331	0.001
	Bray-Curtis	1.919	0.324	0.001
**Between groups (Mwanihana)**	u. UniFrac	2.086	0.258	0.001
	w. UniFrac	3.152	0.344	0.001
	Bray-Curtis	3.128	0.343	0.001

Supplementary Table S4. Permutational ANOVA (PERMANOVA) tests on unweighted (u.) and weighted (w.) UniFrac and Bray-Curtis distance/dissimilarity. Tests were performed using the R package vegan (adonis() function) with 999 permutations.

Group1	Group2	Distance [km]
Ma3	Ma2	1.855
Ma4	Ma1	2.253
Ma2	Ma1	2.393
Mw2	Mw1	2.671
Ma4	Ma2	3.491
Ma3	Ma1	3.635
Ma4	Ma3	5.248
Mw3	Mw2	5.812
Mw3	Ma4	7.293
Mw2	Ma4	7.509
Mw3	Mw1	8.455
Mw1	Ma4	8.591
Mw3	Ma1	8.980
Mw2	Ma1	9.761
Mw2	Ma2	10.371
Mw1	Ma1	10.769
Mw3	Ma2	10.778
Mw1	Ma2	10.793
Mw2	Ma3	12.218
Mw3	Ma3	12.472
Mw1	Ma3	12.550

Supplementary Table S5. Geographical distances (in kilometers) between GPS points of sample sites in Magombera and Mwanihana forests.

Supplementary Table S6 (external file). Predicted functional potential of the gut-associated microbiome in red colobus monkeys (*Procolobus gordonorum*).

Supplementary Table S7 (external file). Sample accession and metadata of 16 samples from Magombera and of 15 from Mwanihana forests.

Supplementary Methods

Fecal sampling

Fecal samples were collected during two dry seasons (January-February and August-September 2013). Social groups moving through the canopy were followed unobtrusively from the ground, and stools were collected once from each social group during a single defecation event (when several individuals defecate simultaneously on the forest floor). Fifteen samples from three Mw social groups, and 16 samples from four Ma groups (mean: 4.4 samples per group; range: 3-7; Fig. 1) were collected. We collected 0.5 g of each feces (representing one individual) in a 15 ml polypropylene tube pre-filled with 5 ml of RNAlater® Stabilization Solution (Ambion, Life Technologies, Monza, Italy), stored at ambient temperature for up to 8 days, shipped to Italy, and stored at -20 °C until DNA extraction. Samples were collected without direct contact or interaction with the animals and under permit approval from the Tanzania Commission for Science and Technology (COSTECH), Tanzania Wildlife Research Institute (TAWIRI) and Tanzania National Parks (TANAPA). Our data collection procedure adhered to the legal requirements and complied with the laws governing wildlife research in Tanzania.

DNA extraction, PCR amplification and pyrosequencing

For each sample, the RNAlater® (Ambion, Life Technologies) was removed from 500 mg aliquots of thawed fecal samples. Genomic DNA was extracted from each sample using the QIAamp DNA Stool Kit (Qiagen, Milano, Italy) following the manufacturers' instructions, quality-assessed by gel electrophoresis and the NanoDrop spectrophotometer (Thermo Fisher, Waltham, MA) and stored at -20 °C until pyrosequencing.
For each sample, we amplified the 16S rRNA gene using the special fusion primer set specific for V1-V3 hypervariable regions (8-Forward: 5'-AGAGTTTGATCMTGGCTCAG-3' and 533-Reverse: 5'-TTACCGCGGCTGCTGGCAC-3'). The forward primer included: the Lib-L primer A sequence (Roche, Branford, CT), the key sequence TCAG, the sample-specific barcode Multiplex Identifier (MID) sequence and the 8-Forward sequence. The reverse primer comprised the Lib-L primer B sequence, the key sequence TCAG and the 533-Reverse sequence. For each sample, a PCR mix of 25 µl was prepared containing 1X PCR buffer, 1.25 U of FastStart High Fidelity polymerase blend (Roche Life Science, Milano, Italy) and dNTPs from the FastStart High Fidelity PCR system (Roche Life Science, Milano, Italy), 0,4 µM of each primer (PRIMM, Milano, Italy) and 10 ng of gDNA.

Thermal cycling consisted of initial denaturation at 95 °C for 5 minutes followed by 35 cycles of denaturation at 95 °C for 30 seconds, annealing at 58 °C for 30 seconds, and extension at 72 °C for 1 minute, with a final extension of 8 minutes at 72 °C.

Library construction and pyrosequencing

The PCR products of the 31 samples (16 from Ma, 15 from Mw forest and three replicates) were analysed by gel electrophoresis and cleaned using the AMPure XP beads kit (Beckman Coulter, Brea, CA, USA) following the manufacturer's instructions; quantified via PCR using the Library quantification kit Roche 454 titanium (KAPA Biosystems, Boston, MA) and pooled in equimolar proportion in a final amplicon library. The 454 pyrosequencing was carried out on the GS FLX+ system using the XL+ chemistry following the manufacturer's recommendations.

Supplementary References

1. Pucci, A. & Rovero, F. Observations on the Food Habits of the Endemic Udzungwa Red Colobus (Piliocolobus gordonorum) from Different Forest Sites in the Udzungwa Mountains, Tanzania. in *Folia Primatol.* **75:406,** (2003).

2. Davies, G. & Oates, J. *Colobine Monkeys: Their Ecology, Behaviour and Evolution*. (Cambridge University Press, 1994).

3. Struhsaker, T. T. *The Red Colobus Monkeys: Variation in Demography, Behavior, and Ecology of Endangered Species*. (Oxford University Press, 2010).

4. Platts, P. J. *et al.* Delimiting tropical mountain ecoregions for conservation. *Environ. Conserv.* **38,** 312–324 (2011).

5. Marshall, A. R. *Ecological report on Magombera forest*. (2008). Available at <http://www.easternarc.or.tz/MagomberaEcologicalReport2008.pdf>

6. Lovett, J. C., Marshall, A. R. & Carr, J. Changes in tropical forest vegetation along an altitudinal gradient in the Udzungwa Mountains National Park, Tanzania. *Afr. J. Ecol.* **44,** 478–490 (2006).

7. Cavanaugh, K. C. *et al.* Carbon storage in tropical forests correlates with taxonomic diversity and functional dominance on a global scale. *Glob. Ecol. Biogeogr.* **23,** 563–573 (2014).
